# Monocyte/macrophage and T-cell infiltrates in peritoneum of patients with ovarian cancer or benign pelvic disease

**DOI:** 10.1186/1479-5876-4-30

**Published:** 2006-07-06

**Authors:** Xipeng Wang, Michael Deavers, Rebecca Patenia, Roland L Bassett, Peter Mueller, Qing Ma, Ena Wang, Ralph S Freedman

**Affiliations:** 1 Department of Obstetrics and Gynecology, Renji Hospital, Shanghai Tiao Tong University, Shanghai, China; 2 Department of Pathology, The University of Texas M. D. Anderson Cancer Center, Houston, Texas, USA; 3 Department of Gynecologic Oncology, The University of Texas M. D. Anderson Cancer Center, Houston, Texas, USA; 4 Department of Biostatistics and Applied Mathematics, The University of Texas M. D. Anderson Cancer Center, Houston, Texas, USA; 5 Department of Blood and Marrow Transplantation, The University of Texas M. D. Anderson Cancer Center, Houston, Texas, USA; 6 Department of Transfusion Medicine, National Institutes of Health, Bethesda, Maryland, USA

## Abstract

**Background:**

We previously showed that tumor-free peritoneum of patients with epithelial ovarian cancer (EOC) exhibited enhanced expression of several inflammatory response genes compared to peritoneum of benign disease. Here, we examined peritoneal inflammatory cell patterns to determine their concordance with selected enhanced genes.

**Methods:**

Expression patterns of selected inflammatory genes were mined from our previously published data base. Bilateral pelvic peritoneal and subjacent stromal specimens were obtained from 20 women with EOC and 7 women with benign pelvic conditions. Sections were first stained by indirect immunoperoxidase and numbers of monocytes/macrophages (MO/MA), T cells, B cells, and NK cells counted. Proportions of CD68+ cells and CD3+ cells that coexpressed MO/MA differentiation factors (CD163, CCR1, CXCR8, VCAM1, and phosphorylated cytosolic phospholipase A_2 _[pcPLA_2_]), which had demonstrated expression in EOC peritoneal samples, were determined by multicolor immunofluorescence.

**Results:**

MO/MA were present on both sides of the pelvic peritoneum in EOC patients, with infiltration of the subjacent stroma and mesothelium. CD68+ MO/MA, the most commonly represented population, and CD3+ T cells were present more often in EOC than in benign pelvic tumors. NK cells, B cells, and granulocytes were rare. CXCL8 (IL-8) and the chemokine receptor CCR1 were coexpressed more frequently on MO/MA than on CD3+ cells contrasting with CD68+/CD163+ cells that coexpressed CXCL8 less often. An important activated enzyme in the eicosanoid pathway, pcPLA_2_, was highly expressed on both CD68+ and CD163+ cells. The adherence molecule Vascular Cell Adhesion Molecule-1 (VCAM1) was expressed on CD31+ endothelial cells and on a proportion of CD68+ MO/MA but rarely on CD3+ cells.

**Conclusion:**

The pelvic peritoneum in EOC exhibits a general pattern of chronic inflammation, represented primarily by differentiated MO/MA, and distinct from that in benign conditions concordant with previous profiling results.

## Background

Epithelial ovarian cancer (EOC) results in 5 year survival rates of only 25–30% for patients with stage III and IV disease [1], contrasting with the 90% survival rates of patients with stage I disease, where notably peritoneal and serosal disease is absent. It is perhaps a paradox that the peritoneum which is organized to protect the integrity of intraabdominal organs by facilitating infiltration of inflammatory cells to sites of injury and infection, might also serve to facilitate the promotion of tumor growth and spread.

As EOC advances and penetrates the capsular layer of the ovary, it also carries the potential to expose the peritoneal surface to tumor-cell secreted products. The peritoneum and its extension, the intestinal serosa, include a vast surface area for transit of inflammatory cells into the abdominal cavity. Its surface mesothelium and submesothelial stroma and structure pose no substantial barriers to inflammatory modulatory cytokines, chemokines and other molecules produced by the tumor or its metastasis, at least to a depth of approximately 1 mm [2]. The stroma consists of a collagen-based matrix, blood vessels, lymphatics, nerve fibers, and rare hematogenous cells [3,4]. Surgery for EOC often reveals changes in the non-tumor-bearing peritoneum such as thickening or edema, enhanced vascular patterns, and soft or firm adhesions [5]. The peritoneum and intestinal serosa may have a florid appearance similar to that found in peritonitis. Despite this evidence of inflammation, the inflammatory process in the peritoneum of patients with EOC has not been adequately described or characterized.

Using a previously validated cDNA microarray platform consisting of 17,500 clones enriched with inflammatory and immunologically relevant genes [6-8], we previously showed that the gene profiles of the pelvic peritoneum in patients with EOC exhibited a pattern consistent with the presence of MO/MA differentiation, activation, and cell survival and that the pattern was different from that of the peritoneum of patients without cancer or that of the tumor itself [9]. Categorizing genes on the basis of annotated gene function led to our observing that genes associated with inflammation were overexpressed in non-tumor bearing peritoneum of patients with ovarian cancer as compared with the peritoneum of patients with benign ovarian tumors.

The purpose of the study reported here was to describe the global pattern of the main inflammatory cell populations in the peritoneum and stroma and to determine whether the magnitude of expression of a limited group of inflammatory genes could be confirmed at the cellular proteomic level in peritoneal tissue and ascites cells.

## Methods

Peritoneal and subjacent stromal biopsy specimens were obtained from 20 patients with EOC and from 7 patients with benign ovarian or other pelvic tumors who underwent surgery at M. D. Anderson Cancer Center according to a protocol approved by the appropriate institutional review board. Demographic characteristics of those patients are shown in Table 1. Biopsy samples were obtained from the peritoneum and from the submesothelial stroma on both sides of the pelvis, approximately 2 cm from the nearest visible tumor deposits, as quickly as possible after the abdominal cavity was accessed. Peritoneal biopsy samples were obtained carefully without prior manipulation of the chosen biopsy sites to minimize artifact induced variability. As controls, specimens were obtained from similar peritoneal sites in consenting subjects who were undergoing pelvic abdominal surgery but who did not have a diagnosis of cancer. The combined thickness of the peritoneal and separately obtained deeper stromal biopsy specimens was estimated at 1–2 millimeters. A technician was present in the operating room to receive and process all biopsy specimens. All specimens were bisected. One portion, for histopathologic, immunohistochemical, and immunofluorescence costaining, was collected and transported to the lab on ice where it was snap-frozen in Polyfreeze Tissue Freezing Medium (Polysciences, Warrington, PA). Another portion, to be used for microarray, was placed in a sterile tube containing 5% dextrose 0.2% sodium chloride solution and transported on ice to the laboratory. The tissue was removed from the saline solution and snap-frozen in a vial with RNAlater (Ambion, Austin, TX) to minimize RNA metabolism and degradation. Subsequently, peritoneal tissue was also obtained from several additional patients for eicosanoid studies. This tissue was placed dry into a sterile tube and snap-frozen in liquid nitrogen in the operating room. All tissues were stored at -80°C. Benign cases included: ovarian fibrothecoma (3), serous cystoadenoma or cystoadenofibroma (3), and ovarian papillary proliferation (1). A gynecologic pathologist (M.D.) reviewed all hematoxylin-and-eosin (H&E) -stained sections from specimens used in this study. Peritoneal specimens showing microscopic tumor involvement were not included in the studies described here.

**Table 1:** Clinical characteristics of the 20 chemo-naive patients with epithelial ovarian cancer

Characteristics	N (%)
Mean Age, years (range)	
Patients with malignant disease (n = 20)	60 (36 – 79)
Patients with benign ovarian disease (n = 7)	64 (47 – 83)
	
EOC Histology	
Serous	10 (50%)
Mucinous	2 (10%)
Endometrioid	2 (10%)
Clear cell	1 (5%)
Mixed	5(25%)
	
Disease Stage	
I – II	2 (10%)
III – IV	18 (90%)
	
Tumor Grade	
I	2 (10%)
II	2 (10%)
III	16 (80%)
	
Surgical Debulking	
Optimal	11 (55%)
Suboptimal	9 (45%)

### Immunohistochemical staining of peritoneal biopsy tissues

#### Indirect immunoperoxidase (IIP) staining

To determine the proportions of infiltrating mononuclear leukocyte populations in the peritoneal stroma, cryopreserved peritoneal biopsy specimens were cut and stained, using an avidin-biotin immunoperoxidase method [10,11]. IIP staining is generally considered more sensitive and specific than H&E for staining and identifying mononuclear leukocyte populations in cryopreserved tissue. Briefly, 6-μm sections of cryopreserved peritoneal tissues were immediately fixed with acetone for 10 minutes, air-dried for 30 minutes, and then kept at 20°C overnight. Sections were then air-dried for another 30 minutes at room temperature and endogenous peroxidase activity was blocked by incubation in 0.3% H_2_O_2 _in PBS for 15 minutes. Sections were then washed three times in PBS, and nonspecific reactions were blocked with 2% normal horse serum for 30 minutes. Sections were then incubated for 2 hours at room temperature with the primary antibodies as follows. For the immunohistochemical analyses, primary antibodies were: Mouse anti-human CD45 leukocyte common antigen [LCA] clones 2B11 + PD7/26, catalog no. M0701, Mo IgG1, 1/400 (DakoCytomation, Carpinteria, CA); mouse anti-human CD3 clone T3-4B5, catalog no. M0756, Mo IgG1, 1/225 (DakoCytomation); mouse monoclonal antibody KP1 to CD68, catalog no. ab955, Mo IgG1, 1/2000 (Abcam, Cambridge, MA); mouse anti-SCLC (CD56, N-CAM) clone 123C3. Mo IgG1, 1/50 (Zymed Labs, San Francisco, CA); and mouse anti-human CD20 clone B-LyI, catalog no. M0774, Mo IgG1 kappa 1/700 (DakoCytomation). Secondary antibodies used were: biotinylated horse anti-mouse IgG (1:200) (Vector Laboratories, Burlingame, CA); Universal LSAB kit/HRP Rabbit/Mouse, catalog no. K0675 (DakoCytomation), and appropriate isotype controls. Optimal conditions for staining with each antibody were determined by using appropriate test tissues. After being washed with PBS three times, sections were incubated with the appropriate secondary antibody for 1 hour at room temperature. Sections were then washed again with PBS and incubated with avidin-biotin peroxidase conjugate (ABC Kit, Vector Laboratories, catalog no. PK6102) at a dilution of 1:100 for 30 minutes at room temperature, after which AEC substrate (Vector Laboratories, catalog no. SK-4200) was added for 10 minutes. Sections were washed with tap water, counterstained with Vector hematoxylin (Vector Laboratories) for 1 minute, and mounted with permanent aqueous mounting medium (Biomeda, Foster City, CA, catalog no. M03) [10].

Coded slides for indirect immunoperoxidase staining were counted in nine areas per tissue section by random field selection, and the number of cells was averaged per 0.08 mm2. Distributions and ratios of MO/MA and T cells were determined by quantitative immunochemical analysis with a Leica DM LB (Leica, Germany) image analyzer equipped with Image Pro Plus software (Media Cybernetics, Silver Springs, MD) [9]. The Image ProPlus software program evaluates random counting of positive cells by using a grid mask in the process menu, and artifacts are removed by using the delete option.

#### Multi antibody immunofluorescence costaining and confocal microscopy of peritoneal biopsy specimens

*In situ *cell populations or subsets were examined by multicolor immunofluorescence costaining to detect surface receptors and certain cytoplasmic proteins that had been identified in our previous transcriptome studies [9,12]. For the experiments described here, CCR1, CXCL8 (IL-8), CD163, and VCAM1 were included for costaining mononuclear leukocytes that expressed CD68+ or CD3+. In some experiments, MO/MA and the CD163+ subset were costained with phosphorylated cytosolic phospholipase 2 (cPLA2), which together with sPLA2 (Group 2a) was overexpressed in peritoneal samples from EOC patients. Freshly cut tissues (6 μm) were fixed in 4% paraformaldehyde for 20 minutes at room temperature, after which sections were washed in PBS, permeabilized with 0.5% Triton 100×, blocked with 5% normal goat serum, and incubated with primary antibodies overnight at 4°C.

#### IIF triple costaining

A sequential staining technique was used for this method as follows: 3 hours incubation with the first primary antibody (red) at RT, overnight incubation with the second primary antibody (blue) at 4°C, and 3 hours incubation with the third primary antibody at RT. Secondary antibodies were incubated with the sections for 1 hour after the incubations with the primary antibodies were complete. Nonspecific binding was blocked by adding 5% normal goat serum for 1 hour. The primary antibodies used were: mouse anti-human CD3 clone T3-4B5, Mo IgG1 kappa, 1:225 dilution, catalog no. M0756 (DakoCytomation); polyclonal rabbit anti-human CD3, 1:100, catalog no. A0452 (DakoCytomation); mouse anti-human CD68, Mo IgG2a, 1:30, catalog no. MCA1815 (Serotec, Raleigh, NC); mouse monoclonal antibody KP1 to CD68, Mo IgG1 kappa, 1:1500, catalog no. ab955 (Abcam); mouse anti-human CD163, Mo IgG1, 1:100, catalog no. MCA1853 (Serotec); mouse anti-humanVCAM1 clone 1.4C3, Mo IgG1 kappa, 1:50, catalog no. M7106 (DakoCytomation); mouse anti-human CCR1, Mo IgG2B, 1:100, catalog no. MAB145 (R&D Systems, Minneapolis, MN); mouse anti-human CD14, Mo IgG2a kappa, catalog no. M0825 (DakoCytomation); polyclonal rabbit anti-human IL-8, 1:5, catalog no. AHC0881 (Biosource, Camarillo, CA); phospho-cPLA2 (Ser 505) antibody (rabbit) #2831, 1:50 (Cell Signaling Technology, Danvers, MA); mouse anti-human CD31 (PECAM-1, Platelet gpIIa Molecule), catalog no. C2383-02, Mo IgG2b, 1:100 (United States Biological, Swampscott, MA); and mouse anti-human cytokeratin clone AE1/AE3, Mo IgG1 kappa, 1:50, catalog no. M3515 (DakoCytomation). The secondary antibodies used depended on the isotype of the primary antibodies, and included Cy2-conjugated (green) AffiniPure goat anti-mouse IgG, Fcγ subclass 1-specific, catalog no. 115–225-205; Cy3-conjugated (red) AffiniPure goat anti-rabbit IgG (H+L), catalog no. 111–165-144; Cy5-conjugated (blue) AffiniPure goat anti-mouse IgG, Fcγ subclass 2b-specific, catalog no. 115–175–207; Cy5-conjugated (blue) AffiniPure goat anti-mouse IgG, Fcγ subclass 2a-specific, catalog no. 115–175–206; Cy3-conjugated (red) AffiniPure goat anti-mouse IgG, Fcγ subclass 2a-specific (minimal cross-reaction with bovine, and rabbit serum proteins), catalog no. 115–165–206 (all from Jackson ImmunoResearch Laboratories, West Grove, PA). Negative controls employed secondary antibodies alone.

Tissue sections were mounted with Slow-Fade Gold Anti-Fade reagent (catalog no. S36936, Molecular Probes) and viewed with a Olympus FV500 laser scanning confocal microscope; images were captured at 400× and 600× magnification by Fluoview software Version 4.3.

#### Statistics

Wilcoxon tests were used to compare the distribution of markers between malignant and benign samples. Paired t-tests were used to compare inflammatory cell numbers from each side of the pelvis and between the superficial and deeper stroma.

## Results and discussion

**Figure 1:**
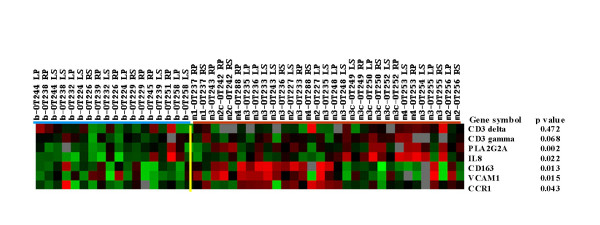
Selected genes expressed at different levels in the peritoneum and stroma of patients with EOC vs in patients with benign pelvic disease. The red bars indicate the malignant phenotype and the blue bars the benign controls. The significance level of each gene expression between benign and malignant phenotypes is presented as P_(t2) _values.

We first examined our data base of more than 50 inflammation-linked genes that have been shown to be expressed differently in malignant and benign peritoneum [9]. The genes in Figure 1 were mined from a database that supported our earlier report on the peritoneal transcriptome [9]. Gene expression levels were generated by centering followed by cluster analysis and displayed as dendrograms (trees) [13]. Here we show expression of several of these genes that encode for the following molecules: CXCL8, a contributor to tumor angiogenesis and leukocyte chemotaxis, CD163 the scavenger molecule associated with MA differentiation, CCR1, a chemokine receptor expressed on different leukocytes, including MA and that binds to multiple CC ligands produced by ovarian cancer cells, and MA, VCAM1, an adherence moledule and ligand for VLA-4 integrin and induced on endothelial cells by tumor necrosis factor (TNFα), interleukin 1α (IL1α) and certain other cytokines. We also detected increased expression of phosphorylated phospholipase A2 (sPLA2), the activated cytosolic form of the protein that releases arachidonic acid from membranes (a critical early step in the eicosanoid pathway) and that can induce CXCL8 (IL-8), IL-6, and CD44 production. Our previous gene profile analysis of the EOC peritoneum suggested that CXCL8 (IL-8) has a central role in these inflammatory cell pathways [9,12]. Analysis of the mined data for both sPLA2 (group 2a) and cPLA2 (group 4a) revealed significant differences in transcript levels, being higher in EOC tumor (n = 8) or peritoneum (n = 10) than in benign peritoneum (n = 5). These differences were statistically significant for cPLA2 and sPLA2 respectively in EOC vs benign peritoneum (P = 0.004) and P = 0.02 and for both cPLA2 and sPLA2 in EOC tumor vs benign peritoneum (P = 0.005 for both). The P values were based on a nonparametric rank-sum test for comparing samples with multiple endpoints [14].

**Figure 2:**
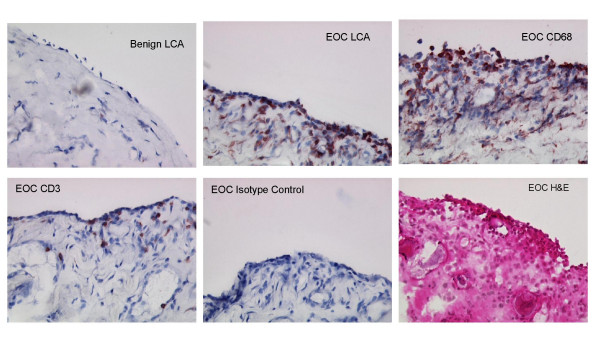
**MO/MA & T-cell infiltration in peritoneum**. Left upper shows peritoneum of patient w/benign fibrothecoma with scant LCA+ leukocytes below the single layer of mesothelium. Remaining 5 panels show tumor-free peritoneum from a patient with EOC. Upper middle shows large number of LCA+ cells; upper right shows large number of CD68+ cells; lower left shows relatively fewer CD3+ cells; lower middle shows negative isotype control; lower right shows H&E. Magnification---200×

Peritoneum biopsy specimens from women with benign pelvic conditions contained fewer leukocytes (Fig. 2) than did peritoneal specimens from patients with EOC, which demonstrated more extensive leukocyte infiltration. Indirect immunoperoxidase staining with anti-CD68 or anti-CD3 monoclonal antibodies revealed that the leukocyte infiltrates consisted of two main populations: MO/MA (CD68+) and T cells (CD3+). LCA was expressed by most peritoneal tissue leukocytes in both malignant and benign conditions. Other cell populations, including granulocytes, B cells, and NK cells, were rare (data not shown). CD68+ (MO/MA) and CD3+ (T-cell) infiltrates were examined in tumor-free peritoneal tissues from 19 of the 20 patients with EOC (one sample was unsuitable for assessment). The mean number of MO/MA per 0.08 mm^2 ^field in the EOC peritoneum was 16.8 and that of T cells was 11.2, as compared with 6.4 MO/MA and 2.6 T cells per field in benign peritoneum (Table 2). MO/MA were substantially more common than were T cells (Table 2) (P = 0.0002 by paired t-test). By contrast, the mean numbers of B cells (CD20+) and NK cells (CD56+) were 4.5 and 1.6 per field in the EOC peritoneum respectively but were rarely detected in benign peritoneum.

**Table 2:** Median numbers of immune cells expressing CD3, CD68, and LCA in peritoneal tissue from patients with EOC or benign ovarian tumors

	CD3 (T cells)			CD68 (MO/MA)			CD45 (LCA)		
	Left	Right	Average	Left	Right	Average	Left	Right	Average

EOC	7.4	9.5	8.7	14.7	15.3	15.3	17.4	18.2	18.1
Benign	1.7	2.0	1.7	7.2	4.1	5.3	3.7	4.7	4.1
P-value	0.012	0.016	0.002	0.038	0.002	<0.001	0.003	0.001	<0.001

Values are expressed as median absolute numbers of cells per 0.08-mm^2 ^field.

MO/MA, monocytes/macrophages; LCA, leukocyte common antigen.

Numbers of CD68+ and CD3+ cells were also examined in paired samples where peritoneal tissues were available from each side of the pelvis. Paired t tests showed no significant differences between the two sides (Table 3). This bilateral presence of MO/MA and T-cell infiltrates in the pelvic peritoneum suggests a spatially generalized distribution pattern rather than a site-specific effect. In the 5 EOC cases in which paired superficial (submesothelial and deeper stroma) samples were available for comparison, more CD3+ cells were found near the mesothelial surface than in the deeper stroma (P = 0.020). CD68+ cells were present in large numbers at both levels.

**Table 3:** The comparison of immune cells with positive CD3, CD68, and LCA markers between left and right sides in patients with EOC.

Marker and Location	No. of Samples		Average Difference (Left – Right)	P Value
CD3				
Peritoneum	15	Median	-0.33	0.89
Stroma	5	Median	-2.40	0.42
				
CD68				
Peritoneum	15	Median	0.57	0.84
Stroma	5	Median	0.10	0.97
				
LCA				
Peritoneum	15	Median	1.27	0.77
Stroma	5	Median	-4.90	0.29

We next examined the MO/MA population, our primary focus for this study, at the cellular proteomic level. The monoclonal antibodies (mAbs) used included those that recognize CD163, CCR1, CXCL8, VCAM1 and cPLA2.

In the present experiments, we examined the two main mononuclear cell populations, CD68+ and CD3+, by indirect immunofluorescence costaining using confocal microscopy, for characteristics that would help to identify functional subsets. Because CD68+ cells were the dominant population in the peritoneum, we reasoned that CD68+ cells (or some subset of those cells) could be a major source of the CXCL8 gene expression product in the peritoneum.

**Figure 3:**
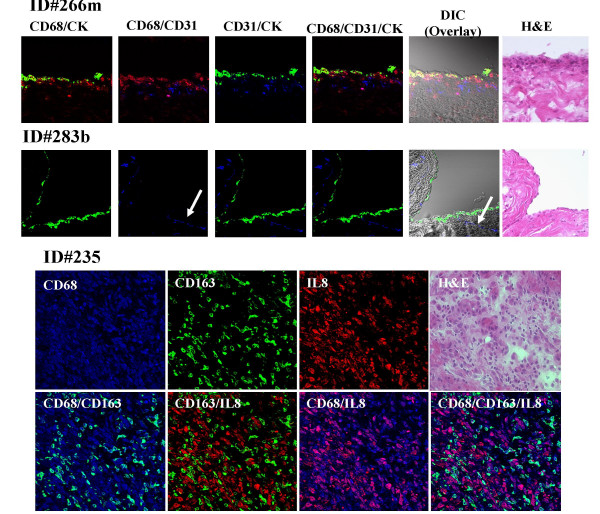
Triple immunofluorescence costaining of frozen right peritoneal tissues were stained with CD68 (red), CD31 (blue), and keratin (CK) (green) antibodies (Rows 1 & 2) Row 1, peritoneal cells from a patient with EOC (ID 266 m) appear yellow from the colocalization of CD68 (red) and CK (green) on some surface mesothelial cells. CD31 staining (blue) indicates endothelial cells just under the mesothelium. Row 2, peritoneal cells from a patient with benign cystic teratoma of the ovary (ID 283b) show prominent staining for keratin in the single cell mesothelial layer but no staining for CD68 staining (red) and positive staining for endothelial cells (blue). Rows 3 and 4, peritoneal cells from patient ID#235 showed colocalization of CD68 (blue) and CD163 (green) appearing cyan color; CD68 (blue) and CXCL8 (red) costaining showed magenta effect and no color changed in CD163+ cells (green). Images were analyzed by confocal laser scanning microscopy (magnification 400×). H&E stained sections are shown for comparison.

Peritoneal biopsy specimens from 7 patients and ascites cytopreparations from 3 patients were tested with triple-antibody staining and evaluated by confocal microscopy. Figure 3 (Row 1) shows surface peritoneum with positive staining for cytokeratin, CD31 (endothelial cells), and CD68. Marked infiltration of CD68+ cells is seen both below and within the surface mesothelium. This is compared with the peritoneum from a patient with a benign condition where only the keratin positive surface mesothelium is shown along with some endothelial cells (Fig. 3, Row 2). The proportions of CD68+ and CD3+ mononuclear leukocytes that coexpress products of certain genes differentially overexpressed in the peritoneal transcriptome profile (Figure 1) are shown in Table 4. Figure 3 and Table 4 show that CXCL8 (IL8), which appears to have a central position in the peritoneal inflammatory transcriptome, was expressed more often on CD68+ cells than on the CD68+ CD163+ subset. CD163 has been identified specifically on a subset of differentiated MO/MA, and might have a role in adaptive immunity [15]. To summarize the important observations, CCR1, the chemokine receptor that binds to a number of different ligands produced in the environment of EOC was expressed on 60.3% (range 44–83%) of CD68+ cells and on only 15.1% (range 0–53%) of CD3+ cells in the peritoneum. Proportions of CCR1 expression were 80.7% and 4% on ascitic CD68+ and CD3+ cells, respectively. The proportion of CD68+ cells or CD68+ CCR1+ cells that coexpressed CXCL8 was also higher than in the CD3+ or CD3+ CCR1+ populations. These results suggest that peritoneal MO/MA might be an important source of CXCL8 in the peritoneal environment of EOC.

**Table 4:** Proportions and mean proportions across samples for CD68+ and CD3+ mononuclear leukocytes expressing CCR1, IL-8, and VCAM1 by confocal microscopy.

**ANTIBODIES**	**PERITO NEAL SPECIM ENS**								**ASCITES**			
	TS-266	TS-265	TS-235	TS-236	TS-267	TS-242	TS-256	M eans	ASC290	ASC288	ASC278	Means

CD68+/CCR1+	65	51	45	44	76	58	83	60.3	87	77	78	80.7
CD68+/IL8+	61	35	38	36	45	66	74	50.7	55	52	42	49.7
CD68+/CCR1+IL8+	66	38	28	30	46	42	37	41	45	35	48	42.7
CD3+/CCR1+	53	19	18	6	2	0	8	15.1	0	10	2	4
CD3+/IL8+	80	45	19	34	7	4	8	28.1	0	10	2	4
CD3+/CCR1+IL8+	47	24	5	11	0	0	0	12.4	0	10	2	4
												
CD68+/CD163+	19	38	31	43	67	39	78	45	30	36	91	52.3
CD163+/IL8+	22	7	0	20	11	45	22	18.1	60	65	26	50.3
CD68+/CD163+IL8+	5	3	0	9	4	22	19	8.9	15	29	32	25.3
												
CD68+/VCAM1+	59	94	68	60	55	4	29	52.7	79	81	26	62
CD68+/CD3+	1	0	1	2	0	6	3	1.9	19	33	15	22.3
CD3+/VCAM1+	4	0	5	4	0	0	12	3.6	38	69	31	46
CD68+/CD3+VCAM1+	1	0	1	0	0	0	2	0.6	16	31	9	18.7
												
CD3/CD68 Ratio	1 to 2	1 to 11	1 to 4	1 to 4	1 to 2	1 to 2	1 to 8		1 to 2	1 to 2	1 to 2	
Disease Stage	III	III	III	III	III	II	II		III	III	III	
Histology	C	E	S/E	S/E	S	S/E	E		S	S	S	

S = serous; E = endometriod; C = clear cell

We found the proportion of peritoneal CD68^+ ^cells that coexpressed CD163 to vary from 19% to 78%; cells that were CD68+CD163+CXCL8+ usually contributed less than 10% of total CD68+ cells (Figure 3, rows 3&4 & Table 4). In the EOC peritoneum, though a higher proportion were present in ascites, CD68+ cells were more often detected within the surface layer of the mesothelium than were CD163+ cells, which seemed to be concentrated below the mesothelial surface. However, in benign conditions, CD163+ cells, though present in smaller numbers, seemed to be more broadly distributed under the mesothelium. The antibody to CD68 used here recognizes a lysozyme marker that can sometimes be coexpressed by keratin-positive epithelial cells [16]. Some large lymphoblastoid T-cells may also coexpress CD68, as suggested by the presence of large ascitic mononuclear cells showing surface staining for CD3 and cytoplasmic staining for the CD68 antigen. CD3 costaining with CD68^+ ^or with CD163+ was rarely observed in the peritoneum, supporting the specificity of the antibody staining for T cells, total MO/MA, and the CD163+ MO/MA subset. CXCL8 was expressed on the surface epithelium in both malignant and benign conditions (data not shown), suggesting that CXCL8, even at low levels, may have a functional role in the absence of cancer.

**Figure 4:**
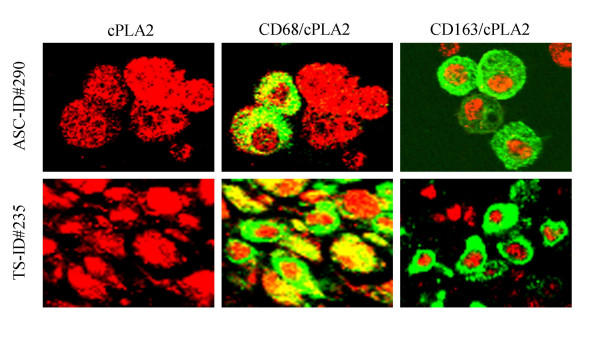
Stained cytospin preps of mononuclear leukocytes isolated from ascitic fluid on F:H density cushion (patient ID 290; top row) or from frozen tissue (patient ID 235; bottom row) were fixed with 4% paraformaldehyde and double-stained with phospho-cPLA2 (Ser505) antibody (red) and CD68 (green) or CD163 (green). Indirect immunofluorescence showed pcPLA2 in both the nucleus and cytoplasm, and results were similar in the cells from ascites and those from tissue. Cells costained for CD68 and cPLA2 showed colocalization of both markers in the cytoplasm and in the nucleus (yellow). However, in CD163+ cells, cPLA2 was seen only in relation to the nucleus.

VCAM1, an important adhesion molecule, was also expressed primarily by CD68^+ ^cells (53%) and by CD31^+ ^endothelial cells but only infrequently by CD3^+ ^cells (3.6%) (Table 4). Finally, CD68^+ ^cells and the CD163+ subset in both peritoneal and ascitic cells also expressed substantial amounts of pcPLA2 (Figure 4). However, the pattern of pcPLA2 expression both in the peritoneum and in ascitic cells seemed to differ between the total population of CD68^+ ^cells and the CD163^+ ^cells; CD68+ cells showed both cytoplasmic and nuclear staining, and CD163+ cells showed costaining of pcPLA2 only in the nucleus.

## Conclusion

Collectively, our results represent the first steps in showing that inflammatory cells have a spatially generalized distribution pattern in the pelvic peritoneum of EOC and that the inflammatory cell subsets are both quantitatively and qualitatively different from the patterns typical of benign pelvic disease. These findings complement those of our previous study of the EOC peritoneal transcriptome [9] and could suggest a common biologic effect. The inflammatory cell infiltrates in cancer could contribute to antitumor effects or, conversely, promote invasion and metastasis. Our previous studies [17,18] suggest that ascitic MO/MA, representing a substantial proportion of the intraperitoneal inflammatory cell environment, include cells that exhibit defective Fcγ R [18]mediated activity or mediate T cell suppressor functions[17]. Here we showed that tissue from nontumor-involved peritoneum in patients with EOC exhibited substantial leukocyte infiltrates in comparison with peritoneal tissues in patients with benign pelvic disease and that the infiltrate consisted mainly of MO/MA and, to a lesser extent, T cells. Other cells (NK [CD56+] and B cells [CD20+]) were found, but in much lower numbers. These results lead us to speculate that MO/MA and T-cell infiltrates in the peritoneum were responding to a general migration stimulating effect; given their proximity to the peritoneal cavity, the large numbers of MO/MA and T cells found in ascitic fluid could well have originated from cells that had migrated into the submesothelial stroma from an extensive network of small capillary vessels, facilitated by expression of adhesion molecules in the capillary endothelium. These views are consistent with the work of Alberto Mantovani [19,20] who has demonstrated the effect of tumor cell products on the "polarization" of MA. In our studies on the peritoneum, we found substantial numbers of CD68+ cells, and CD68+ CD163+ cells, to be concentrated near the mesothelium. The CD68+ CD163^- ^population in particular appeared more likely to coexpress CXCL8, a proangiogenic chemokine that can influence the migration of different leukocyte populations. The presence of CD68 cells within the mesothelial cell layer also suggests that these cells are in transit to the peritoneal cavity compartment or ascitic fluid.

A number of chemokines might contribute to the migration and activation of leukocytic as well as other cells in the EOC environment. Migration effect is dependent largely on the expression of complementary receptors of ascites for a number of CC or CXC chemokines (named for the arrangement of their first two cysteine residues). As we have shown here and elsewhere [12,21], CXCL8 appears to have a prominent role in the peritoneal and ascitic CD68+ population and specifically in CCR1+ cells. At least nine CC chemokines, many of them associated with EOC [21,22], can serve as ligands for CCR1. Here we found CCR1, also highly expressed in the peritoneal microarray profile, on substantial numbers of peritoneal MO/MA and on some T cells, suggesting that CCR1, could play an important role in migration of certain cell populations that express this receptor.

CXCL8 can be induced by various cytokines, including IL-1, TNFα, IL-3, IL-13, and IL-7, most of which are produced in EOC patients and can be induced H_2_O_2 _and hypoxia. CXCL8 binds to CXCR1 or CXCR2, either of which can be expressed on resting T cells but are not usually on monocytes. We found CXCL8 to be produced on a large proportion of MO/MA and, more variably, on CD3+ cells. Moreover, only a very low proportion (< 10%) of the CD68+CD163+ subset produced CXCL8. CD163 has been linked with IL-10 release in atheromatous disease [23], though in the pig, it has been associated with adaptive immunity [15]. The functional role of CD163+ MO/MA in EOC is yet to be determined.

Elevated levels of the pro-angiogenic chemokine CXCL8 (IL8) have been detected in a variety of tumors, including solid EOC [24] and EOC ascites fluid [25] and may promote tumor growth. Low levels have also been detected in serum of certain normal subjects, and we have observed that the single layer of mesothelium in benign pelvic disease may be positive for this cytokine (data not shown). Unpublished data from the Human Cancer Immunology Research Core Facility of M. D. Anderson Cancer Center, moreover, indicate that 8 of 40 normal donors (all females) had IL-8 levels higher than the lowest standard of 9 pg/ml (Dr. James Reuben, personal communication). These findings suggest that lower levels of CXCL8 might have a physiologic role.

Cells that produce CXCL8 in the peritoneum of patients with EOC might be expected to contribute to elevated levels of CXCL8 in the peritoneal, ascitic, and blood compartments of such patients. CXCL8 is likely to have an important role in the development or spread of EOC; it, along with vascular endothelial growth factor, has been linked with unfavorable prognosis in EOC [26]. The TNF-related apoptosis-inducing ligand (TRAIL) can trigger apoptosis in many malignant cells [27], but CXCL8 has been shown to block TRAIL-induced cell death by converting a TRAIL-sensitive ovarian cancer cell line (OVCAR3) into a TRAIL-resistant one. CXCL8 may also regulate the expression of a member of the mitogen-activated protein kinase superfamily, p38γ [28] and with VEGF contribute to increased endothelial capillary functions. Lysophosphatidic acid (LPA), a phospholipid produced from malignant ovarian epithelium, can enhance the expression of CXCL8 by tumor cells and stimulate EOC cell invasion by enhancing membrane type-1 (MT1) matrix metalloproteinase (MMP) mediated activation of MMP2 [29]. Interestingly, cPLA2, an activated enzyme involved in liberating arachidonic acid from cell membranes and dependent on MAPK-induced phosphorylation [30] was highly expressed in CD68+ cells and the CD68+CD163+ subset. p42/44 and p38 MAPK activation is required prior to translocation to the nucleus [31]. Activation of cPLA2 and other phosphorylates by cytokines in the environment of EOC may contribute to CXCL8 production. Arachidonic acid is the precursor of fatty acid derivatives, including LPA, leukotrienes, prostaglandins, thromboxanes, and other important components of the eicosanoid pathways. LPA activates several biological responses through its binding and activation of G-protein-coupled receptors, and has been detected at elevated levels in the ascites and serum of patients with EOC [32].

We might speculate that chemokines and certain cytokines could be involved in recruiting MO/MA and certain T cells into the submesothelial stroma of the peritoneum, where such cells could contribute to tissue reorganization, tumor cell invasion, angiogenesis, capillary leakage, and the production of ascites.

The cytokines most often detected in serum and ascites of patients with EOC include TNFα, IL-10, IL-6, CSF1 (MCSF), IL-1, and TGFβ isotypes, all of which can be produced by activated MA [17,33-37] or by the tumor cells [10,38]. TNFα and IL-1α enhance the expression of adhesion molecules on endothelial cells, thereby contributing to leukocyte attachment and migration. In our study, VCAM1 was coexpressed on both CD31+ endothelial cells and on MO/MA but not on most CD3+ cells, suggesting that the CD68+ cells and CD31+ endothelial cells in particular might be under the influence of VCAM1-sensitive cytokines released into the peritoneal microenvironment. VCAM1 is upregulated on cytokine stimulated endothelium. Such a release could be an important early step in the migration behavior of MO/MA into the peritoneum and ascitic fluid. The presence of endothelial cells (CD31+) in proximity to and the CD68+ cells either interspersed in or just under the mesothelium, suggest that there is a close spatial relationship of peritoneal MO/MA to ascitic MO/MA.

In summary, this study represents the first description of the inflammatory cell response in the peritoneum of patients with EOC. Our findings here support and complement our previous gene profiling study of the peritoneum [9]. We conclude that peritoneal MA that are separated spatially from tumor cells share several similar phenotypic characteristics and express activated gene products that could have important roles in tumor growth and metastases.
